# Transactions of the Amer. Med. Association

**Published:** 1852-03

**Authors:** 


### Part 2—Reviews and Notices of New Works.

ARTICLE I.

*Transactions of the American Medical Association, Instituted* 1847. Vol. IV., pages 677 octavo ; Philadelphia · Printed for the Association by T. R. & P. G. Collins, 1852.

We are glad to receive this volume in which is made a permanent record of all the important improvements made in our great and growing country for the past year.

In looking over the able reports of the standing committees we are forcibly struck with the rich treasures of science therein presented, which have been gleaned from the various sources of information through which they had been scattered during the year. They are here made accessible and rendered permanently convenient for study and future reference. But the gratification which we feel on this account is marred by the reflection that the series of these reports has been interrupted by the abolition of the standing committees of the Association. And unless again restored we shall hereafter allow an account of our *improvements* to remain scattered through the various channels of information as they were previous to the organization of the Association ; many of them to be lost or only ococcupy a local place. In the notice we gave of the proceedings last summer we expressed our regret for this movement, and the perusal of the reports in the volume before us but strengthens our convictions of the impropriety of the move. We have however, but little doubt that upon realizing the loss of these valuable documents, the Association will retrace this step and speedily re-establish, at least, the more important of the standing committees.

As we gave in the July number of our Journal a pretty full notice of the proceedings of the Association, the minutes of which occupy the first forty-four pages, we proceed to notice the reports and papers that form the remaining portion of the volume before us.

The first of them is the report of the Committee on Medical Sciences by Bennett Dowler, M. D., of New Orleans, chairman. This report, like all of the productions of its distinguished author, is a well written and sensible document. However, on account of its necessary want of originality it falls far short of possessing the interest that attaches to most of his writings.

After his introductory remarks Professor Dowler takes up his subject and discusses it in the following order. 1st., Under the head of Anatomy and Physiology, what has transpired that is new in this country is noticed. 2nd., Illustrations o Cerebral and Spinal Physiology—Monstrosities and Traumatic Lessons. 3d., Medical Chemistry—Pharmacy—Therapeutics—Pathology and Miscellaneous Memorandas ; and 4th, Numerical Medicine.

There are three papers appended to the report, the first by Henry F. Campbell, M. D., of Georgia, reporting a remarkable case of Amaurosis, illustrating the anatomy of the Optic Nerves. The second, being a report on Lead Poisoning made to the Physico-Medical Society of New Orleans, by Drs. Jones, Axson and Riddell.

The third being Miscellaneous Memoranda by the author of the Report.

Next in order comes the able report of the committee on Practical Medicine by Prof. Flint of Buffalo, chairman, which was printed and laid before the Association, a pretty full notice of which we have given in a previous number of the Journa

To the Report there are four pages appended. The first of these is an exceedingly well written report of the Cholera, as it appeared and prevailed in Cincinnati, in the year 1850, by Geo. Mendenhall, M. D. Although the author gives his facts without any arguments on the subject of the communicability of the disease, many of them bear very strongly in favor of the doctrine.

While many cases occurred after the disease became epidemic for which no cause could be made out, its introduction to various parts of the city from boats arriving from New Orleans where the disease prevailed when they left ; and its first prevalence being along the river are facts that should outweigh a very large amount of negative evidence.

The fact that Small Pox prevailed at the same time, and large numbers of cases could not be traced to any source of contagion seems to be a very strong hint that inability to trace all cases of supposed contagious diseases to sources of infection should not weigh much against positive evidence of communication.

It seems strange to us that men of intelligence should continue to seek for local causes for a disease that spreads so regularly from place to place and the invasions of which are at such remote intervals of time as Cholera, when no local circumstance or condition has ever been found to correspond with these facts.

Dr. Mendenhall has done the cause of Science good service by this paper which will add much to his already high reputation.

The second paper is a Report of the Fevers, &c., of New Orleans, by E. D. Fenner, M. D., of that city. It gives a brief but very clear account of the prevalence of Cholera in New Orleans during the year 1850, and of a number of Fevers that prevailed there.

The third is on Dengue compiled from the accounts given by Prof. Dickson and Dr. Campbell of Augusta, Ga.

The fourth is an account of an epidemic which prevailed in Alleghany Co., N. Y., during the autumn and winter of 1850, and 1851, by Dr. R. F. Stevens, of Ceres, N. Y.

The Report on Surgery by Prof. Paul F. Eve, of the University of Nashville, Tenn., is worthy of the high reputation of its distinguished author. But our limits will not permit an analysis of the document.

To it are appended three papers ; the first is a report of six additional cases of Œdematious Laryngitis successfully treated by scarifications of the Epiglottis and Aryteno-Epiglotic Folds. By G. Buck, Jr., M. D., of N. Y., who, it will be recollected, reported his treatment by this method in a former volume of the Transactions.

The second paper is a Table of all of the known Operations of Ovariotomy from 1701 to 1851, comprising 222 cases, with a synoptical history of each case, by Prof. Atlee, of Philadelphia, and is a very valuable table.

The third is on the Surgical statistics of New York Hospital. By Dr. Lente, Rresident Surgeon at the request of John Watson, M. D., attending Surgeon.

The Report with these papers forms a large and most valuable document, a copy of which we are glad to possess.

The Report on Obstetrics by D. Humphreys Storer, M. D. of Boston, is a full compend of the improvements and suggestions in this department of our Science during the year.

We have space only to refer to the remarks of the author upon the "extractor," invented and laid before the Profession by the editor of this Journal at the meeting of the Association in Cincinnati, in 1850.

By these it would appear that the author has formed an unfavorable opinion of the instrument and the arguments used in its favor.

1st. The author thinks the straps liable to injure the child.

2d. He objects to the suggestion that we may apply the instrument before the os-tincea is dilated large enough to allow the head to pass.

3d. He intimates that no cases could occur where delivery wonld be "proper or practicable before labor had commenced."

4th. He conceives the only cases where the Extractor could be advantageously applied would be where labor was protracted on account of deficiency of Uterine action.

5th. He thinks that even here, on account of compression of the head being possible by the forceps, they would be preferable to the Extractor.

As to the straps injuring the head of the child, we can safely say that in no instance have the impressions they make been permanent even where strong and long continued force was necessary to effect delivery. And the substitution of ribbons, made strong for the purpose in place of braids will still farther remove the liability as they will keep their width during the application of force.

In regard to its application before the full dilatation of the os tincea, we would simply remark, that in a case of violent puerperal convulsions occurring in the first stage of labor last summer, we applied the Extractor with facility, when the os tincea was only dilated to the size of two inches in diameter, and secured not only its speedy dilatation by gentle extractive force, but in a short time delivered with entire safety to both mother and child.

In cases of violent hemorrhage before labor commences, and in convulsions also, we conceive it might be altogether "proper and practicable" to induce labor and delivery. And we have no doubt that in such cases the "Extractor" will be found to be applicable, gentle traction of the head against the os tincea and the influence of the straps upon it exciting dilatation and bringing on labor. But as this would be a new practice and is yet untried, of course we can only give our opinion upon its philosophy.

Evidently we have failed to convey to the author of the Report, in our description of the instrument, a full understanding of its character and applicability, or his fourth conclusion would not have been made.

The compression of the head by the forceps, we regard as one of the most serious objections to the instrument. First, because the compression necessary to prevent the instrument from slipping off where much extractive force is required is liable to kill the child. And secondly,because the diminution of one diameter of the head by compression with the forceps, is attended by an increase of the diameter that is transverse to it. And the diameter included between the blades of the forceps, the bi-parectal,being shorter already, than the others, of course compression will increase instead of diminishing the difficulty. Of course neither Dr. Storer nor any other good practitioner would advise the application of the forceps upon the face and occiput for the purpose of delivery ; much less to make compression for reducing the size of the head in that direction. We confess we are supprised that the author should have brought forward this argument in favor of the forceps in the faces of such an amount of high authority as is in our standard works arrayed against it.

But we shall shortly publish a report of additional cases in which we have used the Extractor,with the opinions of numerous practitioners, who have witnessed its application,and therefore defer any further notice of the subject at present.

The report of the committee on medical Education, by Worthington Hooker, M. D., of Conn., chairman, is an exceedingly well written document, setting forth in the authors usual clear and elegant style, the defects of our system of education, and suggesting remedies for them. The report closes by a series of resolutions, a report on Demonstrative Midwifery, and a correspondence between Dr. Bowditch and Prof. Horsford of Boston, upon the subject of Practical Chemistry.

The Report on Medical Literature, by Prof. T. Reyburn of St. Louis, chairman, is a well written, full, candid, and truthful exposition of the condition of our Medical Literature. The author refrains from that criticism, in reference to style, which much that is written in our country deserves, lest he might dampen the ardor of inexperienced writers and induce them to with-hold valuable contributions of cases, facts, and opinions from the world.

After the Reports of the Commitree on Publication and of the Treasurer, the Report on Hygiene, by P. C. Gaillard, M. D., of Charleston, S. C., chairman, follows.

This Report discusses the subject of heating and ventilating buildings, the system of Meteorological records proposed by the Smithsonian Institute, and the influence of the clergy in giving currency to quackery.

A table of the mortality of Chicago, by Prof. Herrick, is appended.

The admirable prize Essay on the Corpus Luteum of Menstration and Pregnancy, by John C. Dalton, Jr., M. D., of Boston; now Prof. in the University of Buffalo, with its beautiful colored plates, is the last document in the volume.

After a brief history of the observations that had been made upon the Corpus Luteum previously, the author details his own very interesting observations and experiments :

First: Upon the Corpus Luteum of Menstration. Under this head are reported eleven cases, in which the Corpus Luteum was observed in the human subject at different periods of its developement:

Secondly: Upon the Corpus Luteum of pregnancy in the human female, in which eighteen observations were made.

Thirdly: Upon twenty-six cases in inferior animals.

There are four beautiful colored plates representing the appearances of the ovary with its corpus luteum, graffian visicle, &c., &c. The execution of these plates must have been quite expensive, although they are lithographed, on account of the care necessary in coloring and shading them properly.

With this brief notice of the volume, we must draw our article to a close, only regretting our want of space for a full analysis of the more important documents contained in it.

We regard it as decidedly the best volume that has yet emanated from the Association, nearly every paper being filled with important and interesting matter.

We are sorry the index is so imperfect, as it makes it quite difficult to make references to different parts of the volume.

In looking over the formidable array of special Committees that are to report at the next meeting, a large number of whose reports will be upon the epidemics of different States, and will consequently present a great deal of sameness and a large amount of dry detail of Topography, and Statistics, we can but fear that the next meeting of the Association will have a long and tedious session, and the next volume of the transactions be a far less interesting and valuable one than this before us.

We are very sorry that the entire edition of this volume excepting the copies already disposed of, with the surplus numbers of the preceding volumes were consumed in a late fire in Philadelphia, so that copies of neither can be obtained.

